# Early Onset Fetal Growth Restriction: Does Path to Diagnosis Impact Outcomes and Pathology?

**DOI:** 10.33696/gynaecology.1.002

**Published:** 2020

**Authors:** Brian Burnett, Linda Street, Kristen Quinn, Jeff M. Denney

**Affiliations:** 1 Wake Forest University School of Medicine, Department of Obstetrics & Gynecology, Section on Maternal-Fetal Medicine, Winston-Salem, NC, USA; 2 Medical College of Georgia at Augusta University, Department of Obstetrics & Gynecology, Division of Maternal-Fetal Medicine, Augusta, GA, USA

**Keywords:** IUGR, Preterm delivery, Indicated preterm birth, Fetal growth restriction

## Abstract

**Objective:**

To evaluate demographics and outcomes of maternal-fetal pairs in early onset fetal growth restriction (FGR) requiring delivery prior to 34 weeks’ gestation based on ultrasound indication leading to diagnosis.

**Study Design:**

This is a descriptive study of maternal-fetal pairs with early FGR diagnosed prior to 30 weeks’ gestation and delivering between 22w0d and 34w0d under the care of Wake Forest University Perinatology 01/2012–12/2016. Serial ultrasounds to assess fetal growth and umbilical artery flow Doppler velocimetry were evaluated. Pairs were dichotomized into those with maternal comorbidities leading to ultrasound diagnosis, and those with ultrasound indicated only by appreciation of uterine size less than dates on exam. Patient characteristics and outcomes were tracked. Univariate and multivariate analyses were performed as appropriate.

**Results:**

56 pregnancies were identified with FGR prior to 30 weeks and subsequent delivery prior to 34 weeks. Common comorbidities present in the group with maternal comorbidities included chronic hypertension (30.5%), hypertensive disorders of pregnancy (36.1%), preexisting diabetes (13.9%), gestational diabetes (5.6%). None of the women in the S<D group developed hypertensive disorders of pregnancy or GDM. Other background characteristics were similar. Pregnancies evaluated for size less than dates were diagnosed on average 3 weeks later in gestation, had higher incidence of reverse end diastolic flow on Doppler evaluation both at diagnosis (80% vs. 22.9%, p=0.01, OR 0.08 (<0.01,0.74) and were more likely to be delivered for an urgent indication. Both groups of babies had similar survival to discharge rates and length of stay in the NICU. A subanalysis evaluating only babies with abnormal Doppler studies showed a shorter diagnosis to delivery interval and continued to show increased risk of urgent delivery due to fetal status in those pregnancies diagnosed based on size<dates.

**Conclusion:**

Women measuring size less than dates in the mid-trimester should be evaluated by ultrasound without delays. Early FGR carries a high mortality rate in all cases and in our pilot data, women measuring small were diagnosed later with fetal growth restriction and may represent a severe phenotype with poor fetal-placental circulation. These pregnancies often met criteria for urgent delivery in a short time frame, especially if abnormal umbilical artery Doppler velocimetry was noted.

## Introduction

The etiology of fetal growth restriction is rooted in inadequate maternal-placental vascular malperfusion (MVM) of the placenta. Risk factors for MVM are broad and include maternal, fetal, and placental antecedent determinants. These may include maternal disorders including substance use and abuse, maternal nutrition, multiple gestations, teratogenic exposure, or infection. Fetal genetic and/or structural disorders also play a role [1]. While placental disorders such as umbilical cord abnormalities play a role, one undeniable over-arching biological influence is that of inadequate decidualization as demonstrated in human pregnancy as well as in animal models replicating adverse pregnancy outcomes, namely preeclampsia and growth restriction [1–4].

Inadequate decidualization is the most common pathology associated with fetal growth restriction; this process is characterized by lack of removal of the muscular wall of spiral arterioles effectively decreasing capacitance of blood flow across the uteroplacental interface [3]. Increased resistance of blood flow across the uteroplacental interface is a set up for poor placental perfusion, leading to focal infarcts, thromboses, and fibrin deposition within the placental cotyledons [2–4]. However, the obstetrician effectively sees the placental findings long after it’s relevant with respect to delivery timing—on a placental pathology report days after delivery.

Placental perfusion may be assessed via Doppler measurements of blood flow between the placenta and fetus and enables the clinician to reliably discern which fetuses are highest risk for adverse outcomes as based on resistance of blood flow being pumped from the fetus out to the placenta via the umbilical artery [5,6]. Doppler velocimetry of the umbilical artery is well documented in the literature as an important means to evaluate blood flow between the placenta and fetus [7].

Correlating fetal biometry with assessment of the velocity of systolic to diastolic flow in the umbilical artery enables the obstetrician to identify not only increased resistance of flow but, more importantly, which fetuses face risk for uteroplacental insufficiency and in effect face risk for restriction of growth potential, oxygenation of tissues, and ultimately either hypoxic injury or in utero fetal demise (IUFD) [5–7].

Our group set out to evaluate maternal-fetal pairs who were diagnosed with early onset fetal growth restriction diagnosed prior to 30 completed weeks’ gestational age; specifically, those with maternal comorbidities (ie, hypertensive disorders of pregnancy, pre-existing or gestational diabetes mellitus) leading to ultrasound diagnosis versus those referred for clinical suspicion of growth restriction (ie, following identification as uterine size less than dates). Moreover, our objective was to provide additional insight for risk stratification by maternal medical history in context of severe early growth restriction to predict which pregnancies would be most likely to have MVM across the uteroplacental interface and within the placenta sufficient to lead to either emergent delivery or very severely preterm deliveries as a result of lack of appropriate interval growth paired with severely abnormal fetal-placental blood flow.

## Materials and Methods

This is a descriptive study of maternal-fetal pairs with fetal growth restriction diagnosed prior to 30 weeks’ gestation at Wake Forest University School of Medicine, Department of Obstetrics and Gynecology, Section on Maternal-Fetal Medicine (perinatology, WFUP) in Winston-Salem, North Carolina from January 2012 to December 2016. Wake Forest Institutional Review Board approval was obtained for the research protocol. After approval by the Institutional Review Board and the Wake Forest Clinical Translational Science Institute (CTSI), funding for data collection and project work were obtained through the National Center for Advancing Translational Sciences (NCATS), National Institutes of Health, through Grant Award Number UL1TR001420. Likewise, this study was sponsored by Wake Forest University School of Medicine, Department of Obstetrics & Gynecology, Section for Maternal-Fetal Medicine. Study data were collected and managed using REDCap electronic data capture tools hosted at Wake Forest School of Medicine.

Inclusion criteria were diagnosis and/or confirmation of fetal growth restriction in one of our four WFUP ultrasound units (Comprehensive Fetal Care Center, Downtown Health Plaza, Prenatal Assessment Center of Forsyth Medical Center, or Clemmons Medical Center). Patients were required to undergo confirmation of dates by ultrasound prior to 20 weeks’ gestational age. Serial ultrasound to assess fetal growth and umbilical artery flow Doppler velocimetry were evaluated following the diagnosis of growth restriction.

Following identification of intrauterine growth restriction (IUGR) prior to 30 weeks, patients were maintained on a prospective list for subsequent retrospective review (ie, clinicians were blinded to candidacy for the study and management was by our academic practice’s routine adherence to American College of Obstetrics and Gynecology (ACOG) Practice Bulletin [6], meaning no alteration or intervention in patient care resulted from the study). If delivery occurred between 22 weeks 0 days (viability) and 33 weeks 6 days (prior to late preterm period), subjects were placed onto a list for chart abstraction and review.

To limit transfer bias, study data were collected and managed using REDCap electronic data capture tools hosted at Wake Forest School of Medicine. REDCap (Research Electronic Data Capture) is a secure, web-based application designed to support data capture for research studies, providing 1) an intuitive interface for validated data entry; 2) audit trails for tracking data manipulation and export procedures; 3) automated export procedures for seamless data downloads to common statistical packages; and 4) procedures for importing data from external sources [8].

Selection bias was minimized by including all singleton IUGR fetuses with delivery 22–33 6/7 weeks’ gestation without identification of a fetal anomaly/suspected genetic abnormality or abnormal placentation (eg, exclusion for previa or accreta) and delivery at our hospital. Diagnoses and management of IUGR, preeclampsia with and without severe features and gestational hypertension were according to American Congress of Obstetricians and Gynecologists (ACOG) and Society of Maternal-Fetal Medicine (SMFM) endorsed criteria [9]. Pregnancies were dated according to ACOG criteria- using last menstrual period and first ultrasound to generate the best obstetric estimate [10].

Salient maternal demographic variables were collected as follows: age, ethnicity, gravidity, parity, and substance abuse (tobacco, alcohol, recreational drugs). Pertinent gynecologic variables were abstracted and included the following: last menstrual period (LMP), certainty of LMP, and estimated date of confinement (EDC). Relevant medical, psychiatric, and surgical history was recorded. Fetal biometry and Doppler data were abstracted from ultrasound reports. Gestational conditions (e.g., gestational diabetes (GDM), gestational hypertension (GHTN), preeclampsia, preterm labor, and preterm premature rupture of membranes (pPROM) were recorded and validated by ACOG criteria for diagnosis.

Women were excluded for delivery at 34 weeks or later, multiple gestation, fetal anomalies, fetal genetic abnormalities (eg, aneuploidies or syndromes suspected based on ultrasound, karyotype, or cell free fetal DNA results), or abnormal placentation (eg, placenta previa, placenta accreta, placenta increta, or placenta percreta). Following abstraction of relevant variables, maternal-fetal pairs were dichotomized into those with maternal comorbidities leading to ultrasound diagnosis, and those with ultrasound indicated only by appreciation of uterine size less than dates on exam. Review and abstraction chart data was reviewed by the authors who are trained in obstetrics as well as maternal-fetal medicine. All data was validated and cross-checked by the reviewing MFMs (Drs. Street, Quinn, and Denney).

Statistical analyses were performed by exporting data into STATA version 13.0 (College Station, TX). Univariate and multivariate analyses were performed as appropriate. Analyses were conducted using STATA version 13.0 (College Station, Tx) [11]. Ultrasound data and percentiles for gestational age were obtained from our licensed AS-OBGYN ultrasound software package [12]. Baseline characteristics were summarized with frequencies and percentages for categorical data and median plus interquartile ranges for continuous data. Continuous outcome data were evaluated by D’Agostino & Pearson normality test to assess if data were normally distributed and subsequently evaluated with one-way ANOVA for data with a Gaussian distribution or Kruskal-Wallis test for data with a non-Gaussian distribution. Dichotomized data were evaluated with a Chi-square test [13].

## Results

663 pregnancies were identified as intrauterine growth restriction or lagging fetal growth during the study period. Pregnancies were excluded for dating after 20 weeks (n=129), abnormal placentation (n=7 previa/morbidly adherent placenta), delivery for spontaneous preterm labor (n=57), multiple gestation (n=68), delivery not between 22 weeks and 33 weeks 6 days (n=265), and structural defects and/or suspected aneuploidy-genetic syndrome (n=81). There were no stillbirths of IUGR fetuses meeting study criteria under our practice surveillance during the study period. Consequently, only 56 pregnancies were identified that met study criteria for well-dated singleton fetus without suspicion for anomalies/genetic syndrome or abnormal placentation and delivery in the gestational age window of 22 weeks 0 days to 33 weeks 6 days.

**Figure 1:**
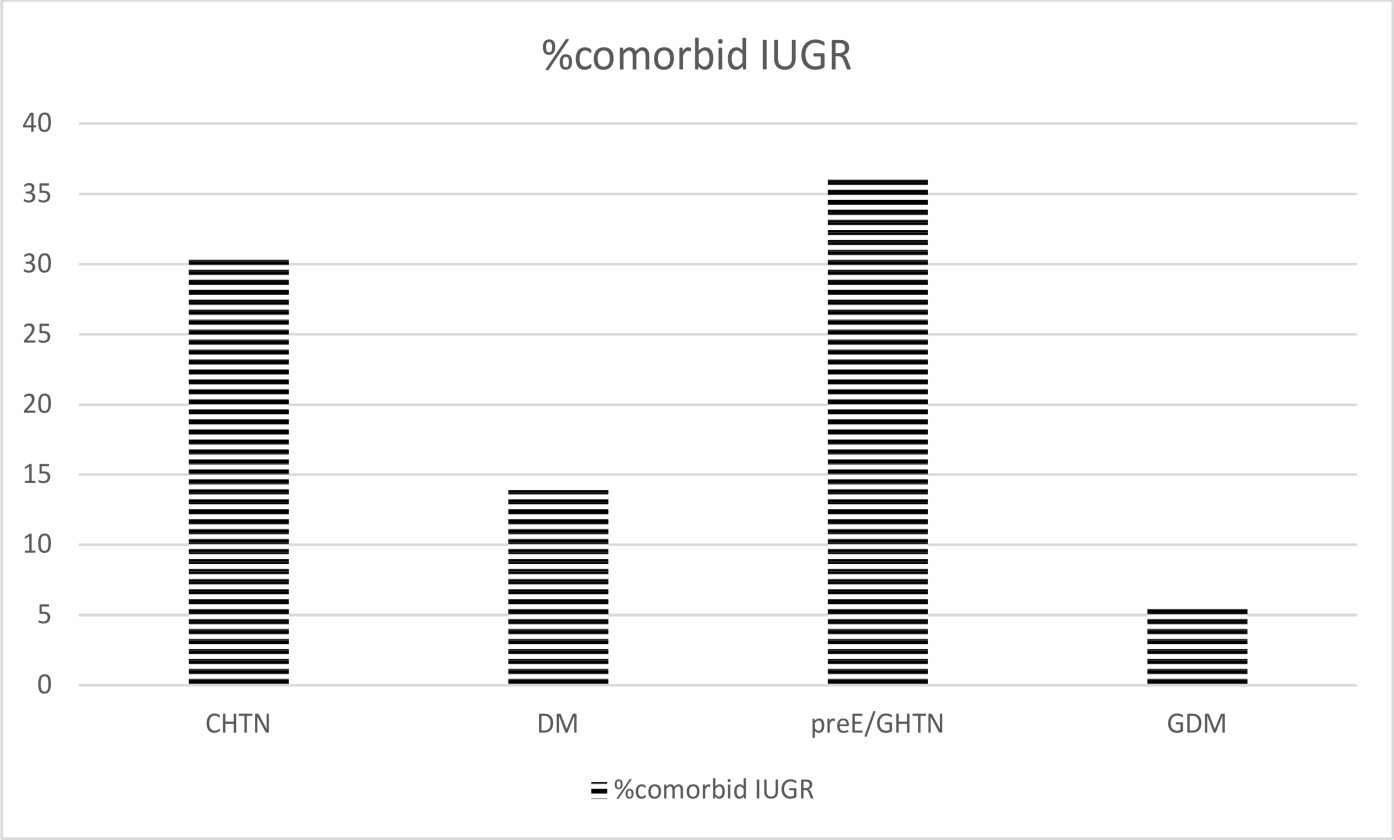
Rate of co-morbidities in the IUGR group complicated by maternal preexisting medical conditions or pregnancy-related medical conditions.

Of the 56 maternal-fetal pairs requiring delivery for IUGR 22w0d to 33w6d in our study, 38 pregnancies were affected by maternal comorbidities and 18 were IUGR fetuses identified for size less than dates (S<D) in otherwise uncomplicated pregnancies. The two groups (S<D vs. maternal comorbidities) had similar demographic characteristic with respect to maternal age (27.0 [6.44] vs. 29.61 [6.95] yrs, p=0.43), gravidity (2.80 [1.92]) vs. 2.92 [1.52], p=0.88), parity (1.4[1.67] vs. 1.03[1.06], p=0.5), body mass index (BMI 28.6 vs 27.9, p=0.61), and tobacco abuse (50% vs. 26%; p=0.09) (Table 1). Common comorbidities present in the group with maternal comorbidities included chronic hypertension (30.5%), hypertensive disorders of pregnancy (36.1%), preexisting diabetes (13.9%), gestational diabetes (5.6%) (Figure 1). None of the women in the S<D group developed hypertensive disorders of pregnancy or GDM. Pregnancies evaluated for size less than dates were diagnosed on average in excess of 3 weeks later in gestation (27.86 weeks [2.92] vs. 24.74 [3.14]; p=0.04). Overall rate of abnormal fetal-placental blood flow as defined by umbilical artery Doppler studies was similarly high in both groups (100% S<D group vs. 84% maternal comorbidity group; p=0.18).

Rates of those delivered for non-reassuring testing in setting of IUGR with simply elevated S/D or intermittent/persistent end diastolic flow at <34 weeks were similar between groups. Both groups had high incidence of requiring delivery by cesarean section (100% S<D vs. 84% maternal comorbidity; p=0.18) (Table 2). However, the S<D group had higher incidence of reverse end diastolic flow on Doppler evaluation at diagnosis (80% vs. 22.9%, p=0.01, OR 0.08 (<0.01,0.74) and were more likely to be delivered for an urgent indication (100% vs. 56%; p=0.02) (Table 2).

**Figure 2:**
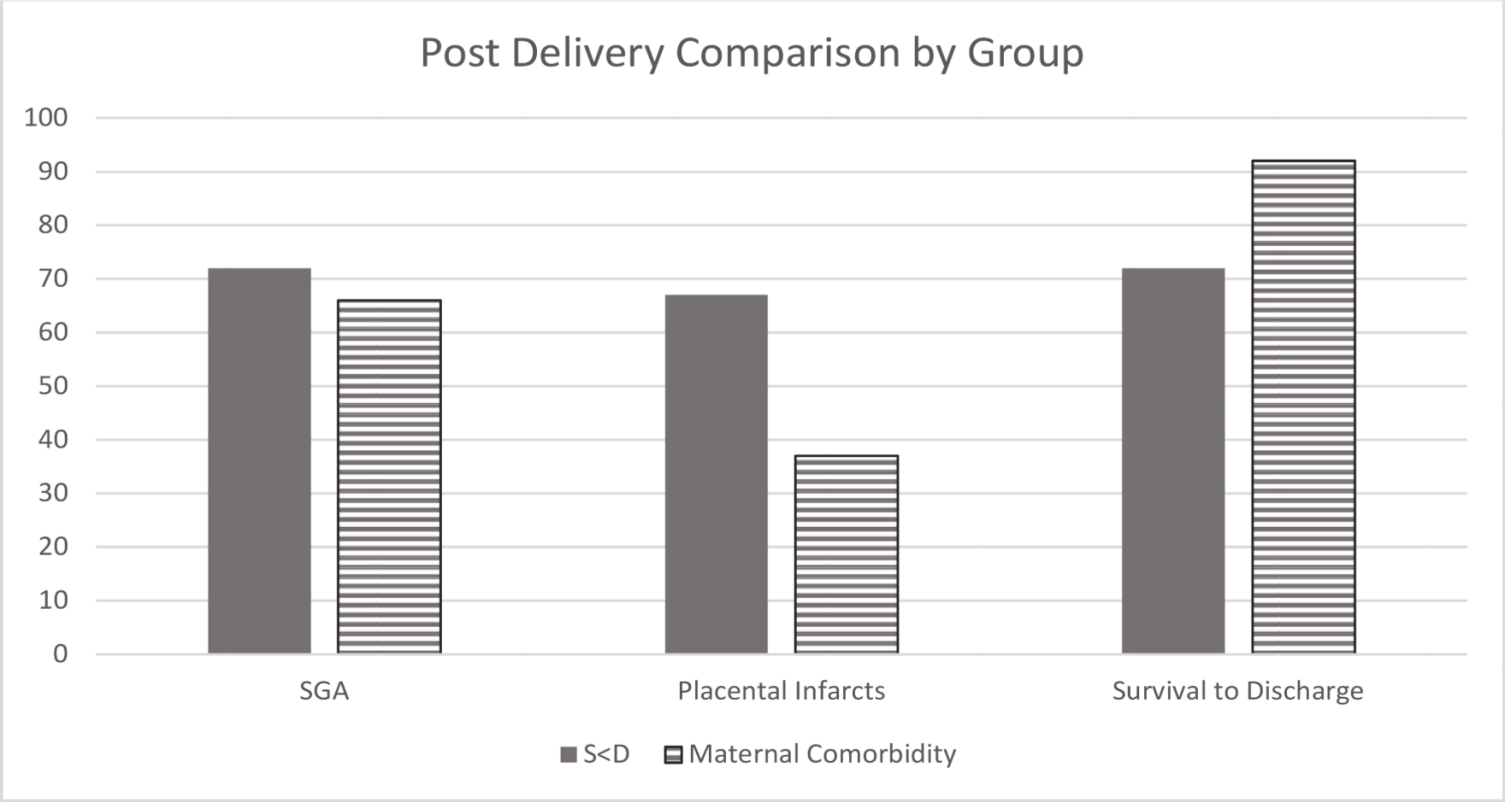
Post delivery data comparison across IUGR groups S<D vs maternal comorbidities.

Both groups of babies were delivered at similar gestational ages (29.46 [2.92] vs. 27.37 [2.60]; p=0.11) and had similar birthweights. The S<D group demonstrated a nonsignificant trend toward less successful survival to discharge rates (72% vs. 92%; p=0.06) S<D group of babies demonstrated similar length of stay in the NICU to the IUGR group with maternal comorbidity (65.65days vs. 67.48 days; p=0.94) (Table 3). Placental morphology varied by group, noting a significant difference in weight (172.5 gm [80.8] vs. 274.6 gm [147.5]; p=0.01) and increased incidence of placental infarcts (67% vs. 37%; p=0.04) (Figure 2).

A subanalysis evaluating only babies with abnormal Doppler studies showed a shorter diagnosis to delivery interval and continued to show increased risk of urgent delivery due to fetal status in those pregnancies diagnosed based on size<dates; (Table 4).

## Conclusions

Early fetal growth restriction carries a high mortality rate in all cases and in our data, women referred from outside practices for fundal heights measuring small were diagnosed later with fetal growth restriction and may represent a severe phenotype with poor fetal-placental circulation. These pregnancies often met criteria for urgent delivery in a short time frame, especially if abnormal umbilical artery Doppler velocimetry was noted. Our data indicate that when our referring providers refer for uterine size measuring less than dates in the mid-trimester, our unit should make every attempt to evaluate these woman-fetus pairs without delay.

In the otherwise low risk patient, measuring size less than dates in the midtrimester appears to be a surrogate for occurrence of an acute placental malperfusion event at higher relative frequency than our population of women with conditions one would classically view as risk factors for poor placental perfusion. Our data demonstrate that pregnancies complicated by referral for S<D have higher incidence of placental infarcts than women with medical comorbidities. However, our data do not address differences in decidualization of the spiral arterioles in the two groups. Our pathologists do not routinely evaluate for this unless specifically requested so this prohibits our group’s ability to evaluate for this difference.

Our data are further limited by inherent subjective component of the diagnosis of a non-reassuring fetal status requiring urgent/emergent delivery. While our hospital and academic obstetrical practice continuously reviews the process and qualifications for indicated delivery, urgent delivery, and emergent delivery, patients in need of either urgent (delivery within 30 minutes) or emergent delivery (delivery within 15 minutes) are oftentimes similar when reviewed retrospectively by committee. For the purposes of this our data analysis, urgent indications (eg, recurrent late decelerations) and emergent indications (eg, fetal bradycardia) are pooled into a singular group of “urgent delivery due to nonreassuring fetal status.” Those indicated for nonreassuring BPP (eg, <4/10 for this group of patients at <34 weeks) or umbilical artery Doppler study waveforms demonstrating persistent reverse end diastolic flow at >29 weeks are categorized as indicated and were typically delivered within 1 an hour after the obstetrician made the decision for delivery and notified nursing and obstetrical anesthesia.

Larger numbers of patients would be needed to evaluate specific differences between patients requiring urgent deliveries versus emergent deliveries. Such data would enable evaluation of what risk factors are associated with need for use of cardiopulmonary resuscitation of the neonate immediately after delivery. Likewise, our dataset is too small to look for differences in rates of neonatal outcomes like respiratory distress syndrome (RDS), intraventricular hemorrhage (IVH), or later outcomes like cerebral palsy (CP). However, our data appear to indicate a significant difference in the urgency in which a child needs to be delivered.

## Figures and Tables

**Table 1:** Maternal demographics by group.

	S<D (18)	Maternal Comorbidity (38)	P value
**Maternal age** (years) Mean (SD)	27.00 (6.44)	29.61 (6.95)	0.43
**Gravidity** Mean (SD)	2.80 (1.92)	2.92 (1.52)	0.88
**Parity** Mean (SD)	1.40 (1.67)	1.03 (1.06)	0.50
**Body mass index** Mean (SD)	28.6 (9.34)	27.9 (9.71)	0.61
**Tobacco abuse**	9/18 (50%)	10/38 (26%)	0.09

**Table 2:** Pregnancy-course with respect to identification and categorization of IUGR by group.

	S<D (18)	Maternal Comorbidity (38)	P value
EGA at diagnosis of FGR (weeks) Mean (SD)	27.86 (2.92)	24.74 (3.14)	0.04
Abnormal umbilical artery (UA) Dopplers at diagnosis	18/18 (100%)	32/38 (84%)	0.18
Elevated UA Dopplers (at diagnosis)	5/18 (28%)	3/38 (8%)	0.06
Intermittent or Persistent Absent EDF (at diagnosis)	5/18 (28%)	17/38 (45%)	0.23
Intermittent or persistent reversed EDF (at diagnosis)	8/18 (44%)	8/38(21%)	0.04
Diagnosis to delivery interval (weeks) Mean (SD)	1.60 (1.01)	2.64 (2.81)	0.42
Cesarean Delivery	18/18 (100%)	32/38 (84%)	0.18
Urgent delivery due to nonreassuring fetal status	18/18 (100%)	22/38 (56%)	0.02

**Table 3:** Neonatal outcome and placental pathology.

	S<D (18)	Maternal Comorbidity (38)	P value
**EGA at delivery** (weeks) Mean (SD)	29.46 (2.92)	27.37 (2.60)	0.11
**Birthweight** (g) Mean (SD)	928 (508)	696 (278)	0.13
**SGA at birth**	13/18 (72%)	25/38 (66%)	0.63
**Placental weight Mean** (SD)	172.5 (80.8)	274.6 (147.5)	0.01
**Placental infarcts**	12/18 (67%)	14/38 (37%)	0.04
**NICU Length of stay in surviving neonates** (days) Mean (SD)	65.65 (54.50)	67.48 (37.61)	0.94
**Neonatal survival to discharge**	13/18 (72%)	35/38 (92%)	0.06

**Table 4:** Pregnancy characteristics and outcomes based on ultrasound indication limited to fetuses with abnormal umbilical artery Doppler studies (subanalysis, n=50).

	S<D (18)	Maternal Comorbidity (38)	P value
**EGA at diagnosis of FGR** (weeks) Mean (SD)	27.25 (2.99)	24.71 (3.22)	0.15
**Tobacco abuse**	9/18 (50%)	8/32 (25%)	0.18
**Maternal ag**e (years) Mean (SD)	26.75 (7.41)	28.87 (6.71)	0.56
**Gravidity** Mean (SD)	2.00 (0.82)	2.87 (1.38)	0.23
**Parity** Mean (SD)	0.75 (0.96)	1.07 (1.11)	0.59
**EGA at delivery** (weeks) Mean (SD)	28.54 (2.39)	26.93 (2.40)	0.22
**Diagnosis to delivery interval** (weeks) Mean (SD)	1.29 (0.85)	4.00 (2.45)	0.04
**Birthweight** (g) Mean (SD)	710 (167)	651 (255)	0.66
**SGA at birth**	13/18 (72%)	28/32 (87.5%)	0.30
**Cesarean Delivery**	18/18 (100%)	29/32 (91%)	0.34
**Urgent delivery due to nonreassuring fetal status**	18/18 (100%)	16/32 (50%)	0.01
**NICU Length of stay in surviving neonates** (days) Mean (SD)	93.00 (38.18)	68.74 (39.97)	0.42
**Neonatal survival to discharge**	2/4 (50%)	22/29 (76%)	0.17

## References

[1] Brosens I, Pijnenborg R, Vercruysse L, Romero R. The “Great Obstetrical Syndromes” are associated with disorders of deep placentation. American Journal of Obstetrics and Gynecology. 2011 3 1;204(3):193–201. 21094932 10.1016/j.ajog.2010.08.009 PMC3369813

[2] Parks WT. Placental hypoxia: the lesions of maternal malperfusion In Seminars in perinatology 2015 Feb 1 (Vol. 39, No. 1, pp. 9–19). WB Saunders. 25511295 10.1053/j.semperi.2014.10.003

[3] Denney JM, Bird C, Gendron-Fitzpatrick A, Sampene E, Bird IM, Shah DM. Renin-angiotensin system transgenic mouse model recapitulates pathophysiology similar to human preeclampsia with renal injury that may be mediated through VEGF. American Journal of Physiology-Renal Physiology. 2017 3 1;312(3):F445–55. 27927648 10.1152/ajprenal.00108.2016

[4] Helfrich BB, Chilukuri N, He H, Cerda SR, Hong X, Wang G, Maternal vascular malperfusion of the placental bed associated with hypertensive disorders in the Boston Birth Cohort. Placenta. 2017 4 1;52:106–13. 28454692 10.1016/j.placenta.2017.02.016 PMC5412713

[5] Ananth CV, Vintzileos AM. Distinguishing pathological from constitutional small for gestational age births in population-based studies. Early Human Development. 2009 10 1;85(10):653–8. 19786331 10.1016/j.earlhumdev.2009.09.004

[6] Galan HL, Grobman W. Fetal Growth Restriction. ACOG Practice bulletin 134, 5 2013.

[7] Berkley E, Chauhan SP, Abuhamad A, Society for Maternal-Fetal Medicine Publications Committee. Doppler assessment of the fetus with intrauterine growth restriction. American Journal of Obstetrics and Gynecology. 2012 4 1;206(4):300–8. 22464066 10.1016/j.ajog.2012.01.022

[8] Harris PA, Taylor R, Thielke R, Payne J, Gonzalez N, Conde JG. A metadata-driven methodology and workflow process for providing translational research informatics support. Journal of Biomedical Informatics. 2009;42(2):377–81. 18929686 10.1016/j.jbi.2008.08.010 PMC2700030

[9] American College of Obstetricians and Gynecologists. ACOG committee opinion no. 560: Medically indicated late-preterm and early-term deliveries. Obstetrics and Gynecology. 2013 4;121(4):908–910. 23635709 10.1097/01.AOG.0000428648.75548.00

[10] American College of Obstetricians and Gynecologists. Committee opinion no 611: method for estimating due date. Obstetrics and Gynecology. 2014;124(4):863–6. 25244460 10.1097/01.AOG.0000454932.15177.be

[11] StataCorp LP. Stata Statistical Software: Release 15.1. College Station, TX: StataCorp LP; Updated November 2018 (perpetual licensure).

[12] AS Medical Information Systems, www.as-software.com Ver 6.9425, copyright 1991–2010.

[13] Koepsell TD, Weiss NS. Epidemiologic Methods. Oxford University Press 2003.

